# Body Mass Index Development from Birth to Early Adolescence; Effect of Perinatal Characteristics and Maternal Migration Background in a Swedish Cohort

**DOI:** 10.1371/journal.pone.0109519

**Published:** 2014-10-10

**Authors:** Mohsen Besharat Pour, Anna Bergström, Matteo Bottai, Jessica Magnusson, Inger Kull, Magnus Wickman, Tahereh Moradi

**Affiliations:** 1 Institute of Environmental Medicine, Division of Epidemiology, Karolinska Institutet, Stockholm, Sweden; 2 Institute of Environmental Medicine, Unit of Biostatistics, Karolinska Institutet, Stockholm, Sweden; 3 Department of Clinical Science and Education, Stockholm South General Hospital, Karolinska Institutet, Stockholm, Sweden; 4 Sachs' Children and Youth Hospital, Stockholm South General Hospital, Stockholm, Sweden; 5 Centre for Epidemiology and Community Medicine, Stockholm County Council, Stockholm, Sweden; Old Dominion University, United States of America

## Abstract

**Background:**

Well documented diversity in risk of developing overweight and obesity between children of immigrant and of native mothers, might be explained by different body mass index (BMI) development trajectories in relation to maternal and perinatal characteristics of offspring.

**Objectives:**

To assess BMI development trajectories among children born to immigrant and to Swedish mothers from birth to adolescence in relation to perinatal characteristics.

**Methods:**

A cohort of 2517 children born in Stockholm during 1994 to 1996 was followed with repeated measurement of height and weight at eleven time points until age 12 years. We estimated changes over time for BMI in relation to maternal and perinatal characteristics of offspring using mixed linear model analysis for repeated measure data.

**Results:**

We observed a significant BMI change over time in children and time interaction with maternal migration status (*P*<0.0001). Estimated BMI over time adjusted for maternal and perinatal characteristics of offspring, showed slower BMI growth before age of 5, followed by an earlier plateau and steeper BMI growth after 5 years among children of immigrant mothers compared with children of Swedish mothers. These differences in BMI growth were more prominent among children with mothers from outside Europe.

**Conclusion:**

Beside reinforcing early childhood as a crucial period in development of overweight, the observed slower BMI development at early childhood among children of immigrants followed by a steeper increase in BMI compared with children of Swedish mothers is important for further studies and for planning of preventive public health programs.

## Introduction

Childhood obesity is a growing epidemic worldwide and recently entitled as a contemporary challenging public health priority by World Health Organization [1]. During the past three decades many western countries including United States have experienced a multiplicative increase in prevalence of childhood obesity [2]. Swedish data shows a similar trend but with a much lower slope as compared with the United States [3]. Despite positive reports indicating flattening childhood obesity trend in Sweden [4], it might be too early to conclude it as in a steady state [5].

The consequences of childhood obesity have a broad spectrum; from psychosocial problems to systemic and metabolic disorders [6], [7]. A large proportion of obese children remain overweight or obese through adulthood, and thus are at increased risk of cardiovascular and metabolic morbidity and mortality [7].

Along rising flow of international migration, concerns have been raised about health status among the growing population of immigrants and their offspring [8], [9]. The result of previous studies indicates a higher risk of overweight and obesity in children born to immigrant parents compared with the native counterparts [10]–[15] and even with children in their home countries [10]. Birth weight, as an indicator for prenatal exposures such as maternal BMI, intrauterine exposure to maternal smoking and maternal diabetes, has been linked to childhood overweight and obesity [16]–[20]. However, existing studies do not support differences in birth weight, as an indicator for different prenatal exposures and intrauterine growth, between children born to immigrant and to native mothers [21]–[24]. To explain diversity in risk of developing overweight and obesity thus, we postulate that there are different BMI development trajectories in relation to maternal and perinatal characteristics between children of immigrant and of native Swedish mothers.

In this longitudinal study using an ongoing cohort of children born in Stockholm during 1992 and 1996, we first, assessed BMI development trajectories among children born to immigrant and to Swedish mothers from birth to 12 years of age in relation to perinatal characteristics. Then we investigated critical time points in BMI changes toward development of overweight and obesity among these children.

## Material and Methods

### Subjects and data collection

A total of 2517 children born in Stockholm were included and followed from birth to the age of 12 years. We retrieved data from the ongoing Swedish prospective birth cohort study BAMSE (Swedish abbreviation for ‘Barn/children Allergy Milieu Stockholm Epidemiology’). Initially, 4089 children born in Stockholm between February 1994 and November 1996 were included in the cohort. Inclusion and exclusion criteria as well as the enrolment process have been described in detail elsewhere [25]. Briefly, at baseline, the parents answered a questionnaire when children were at the age of 2 months. Follow-up questionnaires have been sent to the parents when the children were at the ages of 1, 2, 4, 8 and 12 years. The response rates were 96%, 94%, 91%, 84% and 83%, respectively. Information on maternal country of birth, education, smoking habits during pregnancy and breast feeding were elicited from BAMSE cohort.

When the children were on average 12 years old (age range: 11–14 years), 2,887 parents (71% of the original cohort), gave their consent to collect information on their child's weight and height from school health care register which includes data from child health care centers. In Sweden, almost all children below 2 years of age participate in regular physical examinations at child health care centers and at least one additional examination between 2 and 6 years of age [26], [27]. In child health care centers children's weight and height are measured by trained nurses according to standard national guidelines [27], [28]. When the child starts school, their child health care records are transferred to the school health care. These pre-school health records are then completed with weight and height data routinely measured in 1st, 4th and 7th grade by the school nurses [29].

From the above mentioned sources, available information on weight and height was extracted for 2598 children for 10 predefined ages [6 months (± 2 weeks), 12 months and 18 months (± 4 weeks), 2, 3, 4 and 5 years (±6 months), 7, 10 and 12 years (− 6 to +11 months)]. Then child's BMI was calculated for each predefined ages as weight (kilogram)/height (meter) ^2^.

Information on birth weight and -height, maternal and prenatal characteristics (i.e. maternal BMI at early pregnancy, age, gestational age) and complications during pregnancy (i.e. preeclampsia/eclampsia, diabetes, anemia, placental disease, renal and liver diseases) was obtained from the Swedish Medical Birth Register. The Swedish Medical Birth Register was established 1973 and includes maternal and prenatal information on more than 98% of all infants born in Sweden [30].

The final study population included 2517 children with known maternal country of birth and with information on BMI at least in two time points.

Based on maternal country of birth, children were divided into two groups of immigrant and Swedish mother. We further classified children of immigrant mothers into three sub-groups based on maternal origin: Scandinavian (Finland, Norway, and Denmark), European (excluding Sweden, Finland, Norway, and Denmark) and outside Europe.

### Ethics statement

This study was conducted according to the principles of the Declaration of Helsinki and all procedures involving human subjects/patients were approved by the Regional Board of the Ethical Committee in Stockholm (Dnr: 2011/792-32). All parents gave their written informed consent prior to inclusion of their children in the study.

### Statistical methods

We used chi-square and t-test for categorical and continuous data, respectively, to examine baseline characteristics among children of immigrant and of Swedish mothers in our study population and also to compare baseline characteristics of our study population with BAMSE cohort.

Mixed linear model analysis for repeated measure data was used to predict changes over time for BMI in relation to maternal and perinatal characteristics of offspring including maternal age (year), maternal education (highest attained level of education ≤12 years and>12 years) early pregnancy BMI (kg/m^2^), smoking during pregnancy (at least one cigarette per day any time during pregnancy) (yes/no), pregnancy complications associated with intra uterine growth [31], [32] (preeclampsia/eclampsia, diabetes, anemia, placental disease, renal and liver diseases) (yes/no), weight for gestational age, parity and breast feeding (months). Weight for gestational age was divided into 3 categories based on percentiles derived from Swedish reference curves for normal fetal growth [33]: small for gestational age (SGA, birth weight <10^th^ percentile), large for gestational age (LGA, birth weight>90^th^ percentile) and appropriate for gestational age (AGA, 10^th^ ≤ birth weight ≤ 90^th^ percentile) [34].

To find the best fitted model we used residual log likelihood (-2RLL) for comparison of nested models and Akaike's Information Criterion (AIC) for non-nested models. We applied a polynomial transformation to time variable, child's age, to take into account non-linear changes in BMI during the time. The quadratic (age squared) and cubic (age cube) polynomials provided better model fit compared to crude model. Since the model did not describe rapid variation at about 1 year and 5 years, an additional natural log function of child's age added to the model which improved fit of model also better described the functional changes of child's BMI during time [35]. The final model included maternal migration status as exposure, maternal and perinatal characteristics of offspring as covariates, child's age, age squared, age cube and the natural log of age, as time variables, and interaction between time variables and maternal migration status. In the final model effect of child's age and intercept treated as random variables. The same model was used for analysis of different time points if applicable.

We used SAS version 9.3 (SAS Institute, Cary, NC, USA) for analysis and R version 3.0.2 for graphical presentations. P-values <0.05 were considered to be statistically significant.

## Results

### Demographic characteristics

There were no differences in baseline characteristics between study population and BAMSE cohort with regard to sex, birth weight, gestational age, maternal BMI, maternal smoking during pregnancy, maternal migration status and parity. However, mothers in study population compared with BAMSE cohort had a higher mean age (30.9±4.4 vs. 30.7±4.5 years, *P* = 0.02) as well as higher education (44.1% vs. 41.2% university education, *P* = 0.02) (data not shown).

Table 1 shows maternal and perinatal characteristics of the study population by maternal migration status. In study population 13.3% of the children had foreign born mothers who consist of Scandinavian (30.1%), European (26.8%) and outside Europe (43.1%) origin.

**Table 1:** Maternal and perinatal characteristics of the study population, children born between 1994 and 1996 in Stockholm by maternal migration status.

characteristics		Swedish	Immigrant			
			All immigrant			
				Scandinavian	European	Outside Europe
Number of children		2181	336	101	90	145
Proportion (%)		86.7	13.3	4.0	3.6	5.8
Sex (%)	Girl	49.1	51.8	57.4	55.1	45.8
	Boy	50.9	48.2	42.6	44.9	54.2
Maternal Age in year(±SD^1^)		30.9 (±4.4)	31.0 (±4.7)	31.4 (±4.2)	31.2 (±4.7)	30.7 (±5.1)
Maternal Education (%)	≤12 year	55.6	58.1	71.3**	40.5**	59.7
	>12 year	44.4	41.9	28.7	59.6	40.3
Pre-pregnancy BMI^2^ (kg/m^2^)		22.9 (±3.3)	22.9 (±3.4)	23.4 (±3.5)	22.6 (±3.4)	22.7 (±3.3)
Smoking during pregnancy (%)^3^	Yes	11.9	8.7	13.9	7.9	5.6*
	No	88.1	91.3	86.1	92.1	94.4
Gestational complication (%)	Yes	5.9	3.7	3.0	3.6	4.2
	No	94.1	96.3	97.0	96.4	95.8
Birth weight (gram)		3533 (±572)	3525 (±516.1)	3577 (±484)	3544 (±524)	3476 (±533)
Birth weight (%)^4^	Low BW	3.7	2.5	2	2.3	2.9
	High BW	19.1	17.4	19	17.4	16.6
	Normal BW	77.2	80.1	79	80.2	80.6
Gestational age(week)		39.5 (±1.8)	39.7 (±1.6)	39.8 (±1.5)	39.7 (±1.6)	39.5 (±1.6)
Gestational age (%)^5^	Preterm	9.6	6.4	6.2	9.2	5.0
	Post term	0.5	0.9	2.1	1.2	0.0
	Term	89.9	92.6	91.8	89.7	95
Weight for gestational age^6^	SGA	2.4	1.3	4.2	7.1	10.4
	LGA	4.4	4.7	17.7	11.9	9.6
	AGA	93.2	94.0	78.1	81.0	80.0
Parity (%)	1	55.8	55.4*	52.0	63.2	52.9
	2	33.6	29.1	32.7	20.7	31.4
	>2	10.6	15.6	15.3	16.1	15.7
Breast feeding (months)	Exclusive	5.1 (±2.4)	5.2 (±2.8)	5.1 (±2.6)	4.9 (±2.8)	5.4 (±3.0)
	Total	8.7 (±3.2)	9.1 (±3.7)	9.1 (±3.5)	8.4 (±3.8)	9.5 (±3.8)*

Bolded numbers are statistically significant Comparing immigrant with counterpart Swedish mothers (T-test or chi-square for continuous or categorical variables): * p<0.05, ** p<0.01, *** p<0.001.

Bolded numbers are statistically significant.

1 Standard deviation.

2 Body mass index.

3 Smoking during pregnancy.

4 Low BW = BW<2500 gram; High BW =  BW≥4000 gram; Normal BW =  2500≤BW<4000 gram.

5 Preterm =  delivery<38 weeks; Post term =  delivery> 42 weeks; Term =  delivery between 38–42 weeks of gestation.

6 SGA = small for gestational age; LGA =  large for gestational age; AGA =  appropriate for gestational age.

With exception of having higher parity (>2) which was more prevalent among immigrant mothers there were no differences in gestational age, birth weight, or duration of breast feeding between children of immigrant and of Swedish mother. Further stratification by maternal immigration background revealed that immigrant mothers from other European countries had the lowest proportion of low education and immigrant mothers from outside Europe had the highest proportion on no smokers and the longest duration of breast feeding compared with counterpart Swedish mothers (Table 1). Mean BMI among the children by maternal migration status in different ages are presented in Table S1. Using BMI cut points of International Obesity Task Force (IOTF) [36] we observed that at age 2 years higher proportion of children of Swedish mothers are overweight compared with children of immigrant mothers (10.2% vs. 5.8%, *P* = 0.046) while at age 12 years the overweight proportion is higher among children of immigrant mothers compared with counterpart Swedish children (20.9 vs. 13.8, *P* =  0.001) (data not shown).

### Changes in BMI over time

**Figure 1:**
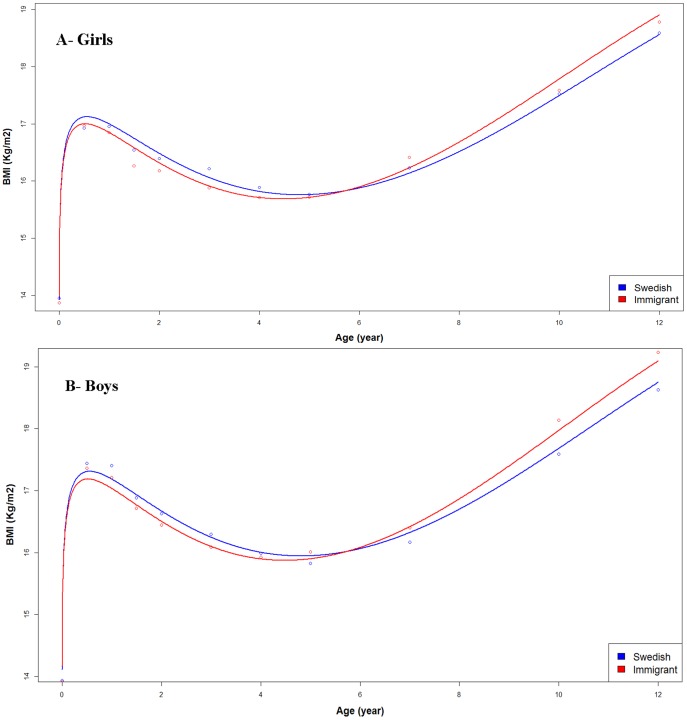
Estimated body mass index (BMI) from birth to 12 years, adjusted for maternal and perinatal characteristics by maternal migration status in girls (A) and boys (B) born between 1994 and 1996 in Stockholm. * Circles represent crude mean BMI.

**Figure 2:**
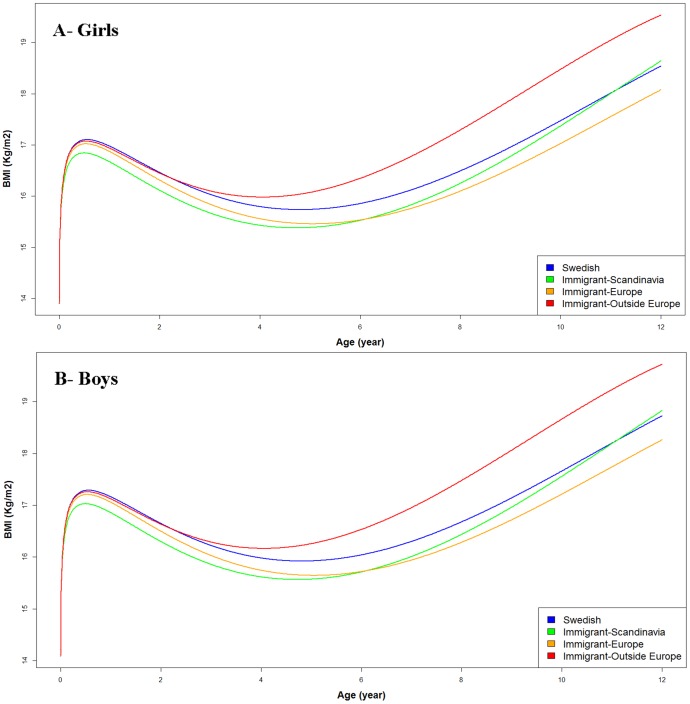
Estimated body mass index (BMI) from birth to 12 years, adjusted for maternal and perinatal characteristics by subgroups of maternal migration status in girls (A) and boys (B) born between 1994 and 1996 in Stockholm.

In the final model, we observed a significant BMI change over time, in multivariable mixed linear model analysis adjusted for maternal and perinatal characteristics and time interactions with maternal migration status (*P* = <0.0001) (Table 2). Graphical presentation of these significant observations illustrated in Figure 1&2 which interpreted as significant BMI changes over time by maternal migration background. We further observed that low BMI at birth was associated with female sex (*P* = <0.0001), having older mothers (*P* = 0.0352), low maternal education (*P* = 0.0035), and small for gestational age (*P* = <0.0001). High maternal BMI during early pregnancy (*P* = 0.0002), and being large for gestational age (*P* = <0.0001) were associated with high BMI at birth (Table 2). We found a significant interaction between maternal migration status and BMI development over time (*P* = <0.0001) (Table 2). Predicted values for BMI development, adjusted for maternal and perinatal characteristics of offspring, showed that while girls and boys of immigrant mothers had slower BMI growth before age of 5 years, the trajectory reached a plateau earlier among them, and BMI grew at a faster rate after age of 5 years than among children of Swedish mother (Figure 1). Analysis confined to age 10 years showed similar results (results not shown).

**Table 2:** Association between maternal and perinatal characteristics and body mass index (BMI) from birth to 12 years of age in the study population, children born between 1994 and 1996 in Stockholm, using conditional mixed model.

parameter			Estimate	Standard Error	Pr> |t|
Intercept			18.1910	0.2442	<0.0001
Maternal migration status	Swedish		0 (ref)	-	-
	Immigrant	Scandinavia	−0.3094	0.1706	0.0697
		Europe	−0.07528	0.2147	0.7259
		Outside Europe	−0.00275	0.1591	0.9862
Sex	Boys		0 (ref)	-	-
	Girls		−0.1838	0.04563	**<0.0001**
Maternal Age (year)^1^			−0.01221	0.005794	**0.0352**
Maternal Education	> 12 years		0 (ref)	-	-
	≤ 12 years		−0.1443	0.04941	**0.0035**
Pre-pregnancy BMI (kg/m^2^)			0.02599	0.007064	**0.0002**
Smoking during pregnancy^2^	No		0 (ref)	-	-
	Yes		0.09235	0.07306	0.2063
Gestational Complication^3^	No		0 (ref)	-	-
	Yes		−0.01182	0.09867	0.9047
Weight for gestational age	AGA^5^		0 (ref)	-	-
	SGA^6^		−0.8891	0.08719	**<0.0001**
	LGA^7^		0.7227	0.06854	**<0.0001**
Parity			0.05863	0.03342	0.0794
Breast feeding (months)^4^			0.009089	0.007266	0.2110
**Time^ 8^**					**<0.0001**
Age			−1.5582	0.02657	
Age*Age			0.1874	0.004776	
Age*Age*Age			−0.00577	0.000251	
Age-log			0.7531	0.006858	
**Time interaction with Migration Status**					**<0.0001**
**- Scandinavians**					**0.0174**
Migration status(S)* Age			0.001563	0.1189	
Migration status(S)* Age*Age			0.001832	0.02145	
Migration status(S)* Age*Age*Age			0.000188	0.001134	
Migration status(S)* Age-log			−0.07410	0.03098	
**- Europeans**					**<0.0001**
Migration status(E)* Age			−0.02744	0.1505	
Migration status(E)* Age*Age			−0.00298	0.02688	
Migration status(E)* Age*Age*Age			0.000234	0.001406	
Migration status(E)* Age-log			−0.01387	0.03857	
**- Outside Europe**					**0.008**
Migration status(O)* Age			−0.0751	0.1115	
Migration status(O)* Age*Age			0.03965	0.0199	
Migration status(O)* Age*Age*Age			−0.0022	0.00104	
Migration status(O)* Age-log			−0.0036	0.02905	

Bold numbers are statistically significant

1 Age at first antenatal clinic visit, almost around week 12−14 of gestation

2 Smoking at least one cigarette per day, at any time during pregnancy

3 Diabetes, preeclampsia and eclampsia, hypertension, anemia, renal disease, liver disease and placental disorders which might affect fetal growth

4 Total breast feeding: number of months that child has been breastfed, both exclusive and partial

5 AGA =  appropriate for gestational age

6 SGA =  small for gestational age

7 LGA =  large for gestational age

8 Time variable in this model is ‘Age’ (year) at weight and height measurement in children. Since the changes in BMI at different ages is not linear or a straight line, different polynomial functions of ‘Age’ has been used to take into account non-linear changes in BMI in different ages. Then, ‘Age’ accounts for linear changes, ‘Age*Age’ accounts for unidirectional curvilinear changes, ‘Age*Age*Age’ accounts for bi-directional curvilinear changes and ‘Age-log’ account for sharp changes, either peak or bottom.

Analysis by subgroups of children to immigrant mothers revealed that change in BMI trajectory toward faster BMI development took place earlier among children with mother born outside Europe (after 2 years) and later among children with mothers from other Scandinavian countries (after 5 years) (Figure 2). Children with European mothers continued with a slower BMI growth through early adolescence compared with children of Swedish mother (Figure 2). Similar results were found when we confined the analysis to children with both parents born outside Sweden (results not shown).

We found no interaction between maternal migration status and maternal education, either overall (P = 0.84) or in subgroup analysis (P = 0.20) (data not presented in table), suggesting similar BMI trajectories for strata of maternal education in relation to migration status.

## Discussion

In this population based cohort study with repeated measurements of height and weight among children from birth to 12 years of age, we observed that despite the comparable birth weight and weight for gestational age, the BMI trajectory over time was different between children of immigrant mothers and of Swedish mothers. Up to age 5 years children of immigrant mother experienced slower BMI development compared with children of Swedish mother. The BMI trajectory reversed after age 5 years and became steeper among children of immigrant mother. BMI changes took place earlier (around age 2 years) and steeper among subgroup of children with mother from outside Europe.

This novel observation is important for further studies and for planning of preventive public health programs. To our knowledge, this study is the first conducted in Sweden to explore the BMI development trajectory among offspring of immigrant mothers and of Swedish mothers from birth to late childhood.

Strengths of our study include the cohort design and repeated anthropometric measurements from birth to age 12 years collected by trained nurses in child health care and school health care centers. Linkage to the high quality Swedish Medical Birth register to retrieve maternal and perinatal characteristics is another important merit of our study. Moreover, using linear mixed model analysis for repeated measurements takes into account correlation of different measurements in each subject as well as variation between and within individuals during time. The overlay of crude mean BMI by maternal migration status at each time point on the fitted BMI trajectory line, given perinatal characteristics, provide an overview of goodness of fit for used model.

The observed steeper BMI development toward higher BMI after age of 5 among children of immigrant mother compared with children of Swedish mother is in line with the results of previous studies reporting higher prevalence or risk of overweight and obesity in offspring of immigrants [10]–[15]. However, to the best of our knowledge, there is no report on slower BMI development before age of 5 years among offspring of immigrants compared with offspring of Swedish mothers. Moreover, most of previous studies among immigrants performed on a cross sectional setting or longitudinal data have been analyzed with conventional methods such as ANOVA or Generalized Estimating Equations assuming constant and equal variation, in other word, an average over time. The few studies that used linear mixed modeling were mostly concerned about difference in generation of immigrants [37], didn’t consider early life characteristics [38], [39] or had a short follow up period [40]. A longitudinal study of children aged 2−12 years showed that proportion of black ethnicity was higher in both early and late onset overweight trajectories than normal weight trajectory [41]. The privilege of our approach is adding time sequence of events to the whole picture. Moreover, we took into consideration perinatal characteristics which are shown to be involved in BMI programming later in life [17], [19], [20].

Our results should also be interpreted under the light of some limitations. First, the nature of our exposure, maternal migration status, makes our exposed group a heterogeneous group in respect to ethnicity, country of birth, genetic and cultural backgrounds and reasons behind migration. We lacked information on genetic and cultural life style factors. Due to lack of statistical power we were not able to perform stratified analysis by specific parental birth country. Nevertheless, we performed analysis in subgroups of children to immigrant mothers based on geographical distance to host country, which might disentangle cultural and lifestyle variations in some degree. This limitation warrants collection of large data with detailed information, specifically on life style factors, in offspring of immigrants.

Furthermore, we defined children of immigrants based on only mother's country of birth due to our concern for perinatal characteristics. It is likely that children with two immigrant parents could differ from children with one immigrant and one Swedish parent in many aspects. The possible misclassification of exposure in our study, however, is probably non-differential which lead to underestimation of the true results, because it is possible for both Swedish and immigrant mothers to have counter-origin husband. Furthermore, this was supported by sensitivity analysis of subgroup of children with both immigrant parents from the same region compared with children of Swedish parents.

We lacked information on child nutrition after weaning until age 5 years; a period were changes in BMI development seem to take place, and could partially explain the observed differences between offspring of immigrant and of Swedish born mothers. We also lacked accurate time for puberty. However, we conducted a sensitivity analysis excluding data on 12 year time point to confine our results to age periods which are unlikely for puberty in both sexes, and yield similar results.

It might be argued that BMI development is part of normal growth and necessarily doesn’t mean overweight or obesity and thus one should use standardized BMI based on age and sex to a reference population. However, direction of BMI changes provides valuable information for preventive purposes and is easy to interpret. In addition it has been shown that BMI changes per se has a better correlation with changes in adiposity especially for assessing adiposity on several time points [42].

Despite the association of perinatal characteristics on programming of BMI development later in life, given controlling for these characteristics, our results suggest that they cannot fully explain the disparity in BMI development in children of immigrant mothers compared with children of Swedish mothers. Nevertheless, our results reinforce early childhood as a crucial period in development of overweight and obesity later in life, a period which could be influenced mostly by cultural, behavioral and life style factors especially at the family level. This finding is important for public health purpose to plan effective preventive programs with regards to critical period and vulnerable groups to combat childhood overweight and obesity later in life and calls for collection of such data in focused groups of offspring of immigrants.

## Supporting Information

Table S1 **Mean body mass index (and standard deviation) in the study population, children born between 1994 and 1996 in Stockholm, by maternal migration status and age.** Mean body mass index was significantly different in immigrant children compared with Swedish children (t-test): * p<0.05, ** p<0.01, *** p<0.001.
(PDF)
