# Supplementary material for: Body Mass Index Development from Birth to Early Adolescence; Effect of Perinatal Characteristics and Maternal Migration Background in a Swedish Cohort
Source: PLoS One. 2014 Oct 10;9(10):e109519. doi: 10.1371/journal.pone.0109519 (PMC4193784; doi:10.1371/journal.pone.0109519)
Supplement: Table S1 — Mean body mass index (and standard deviation) in the study population, children born between 1994 and 1996 in Stockholm, by maternal migration status and age. Mean body mass index was significantly different in immigrant children compared with Swedish children (t-test): * p<0.05, ** p<0.01, *** p<0.001. (PDF) [file pone.0109519.s001.pdf]

Table S1. Mean body mass index (and standard deviation) in the study population, children born between 1994 and 1996 in Stockholm, by maternal migration status and age

Age (year)	Number of children			**Swedish**	**Immigrant**			
					**All Immigrant**			
	Total	Swedish	Immigrant			**Scandinavian**	**European**	**Outside Europe**
0	2398	2086	312	13.93 (1.50)	13.92 (1.36)	14.11 (1.20)	13.83 (1.40)	13.85 (1.43)
0.5	2228	1937	291	17.19 (1.64)	17.14 (1.62)	17.05 (1.68)	17.10 (1.29)	17.24 (1.75)
1	2205	1915	290	17.18 (1.38)	16.99 (1.43)*	16.95 (1.40)	17.07 (1.22)	16.97 (1.55)
1.5	2129	1849	280	16.70 (1.35)	16.47 (1.34)**	16.43 (1.33)	16.46 (1.19)	16.50 (1.44)
2	1486	1280	206	16.50 (1.34)	16.28 (1.23)*	16.15 (1.17)*	16.55 (1.19)	16.25 (1.29)
3	1235	1064	171	16.23 (1.38)	16.00 (1.31)*	15.92 (1.02)*	15.83 (1.11)	16.17 (1.60)
4	2204	1912	292	15.92 (1.30)	15.84 (1.33)	15.71 (1.01)	15.78 (1.31)	15.98 (1.52)
5	2147	1858	289	15.80 (1.50)	15.89 (1.68)	15.55 (1.34)	15.70 (1.37)	16.25 (1.99)*
7	2404	2088	316	16.20 (1.88)	16.48 (2.15)*	16.13 (1.80)	16.27 (1.73)	16.85 (2.53)**
10	2173	1883	290	17.56 (2.46)	17.96 (2.66)*	17.79 (2.72)	17.35 (2.15)	18.42 (2.83)***
12	2190	1884	306	18.62 (2.70)	19.12 (3.11)**	18.95 (3.44)	18.62 (2.47)	19.54 (3.21)***
Total	2517	2181	336	2181	336	101	90	145

Mean body mass index was significantly different in immigrant children compared with Swedish children (t-test): * $p<0.05$, ** $p<0.01$, *** $p<0.001$.
